# ASDmiR: A Stepwise Method to Uncover miRNA Regulation Related to Autism Spectrum Disorder

**DOI:** 10.3389/fgene.2020.562971

**Published:** 2020-10-14

**Authors:** Chenchen Xiong, Shaoping Sun, Weili Jiang, Lei Ma, Junpeng Zhang

**Affiliations:** ^1^Faculty of Information Engineering and Automation, Kunming University of Science and Technology, Kunming, China; ^2^Department of Medical Engineering, People’s Hospital of Yuxi City, Yuxi, China; ^3^School of Engineering, Dali University, Dali, China

**Keywords:** miRNA, lncRNA, mRNA, miRNA regulation, autism spectrum disorder

## Abstract

Autism spectrum disorder (ASD) is a class of neurodevelopmental disorders characterized by genetic and environmental risk factors. The pathogenesis of ASD has a strong genetic basis, consisting of rare *de novo* or inherited variants among a variety of multiple molecules. Previous studies have shown that microRNAs (miRNAs) are involved in neurogenesis and brain development and are closely associated with the pathogenesis of ASD. However, the regulatory mechanisms of miRNAs in ASD are largely unclear. In this work, we present a stepwise method, ASDmiR, for the identification of underlying pathogenic genes, networks, and modules associated with ASD. First, we conduct a comparison study on 12 miRNA target prediction methods by using the matched miRNA, lncRNA, and mRNA expression data in ASD. In terms of the number of experimentally confirmed miRNA–target interactions predicted by each method, we choose the best method for identifying miRNA–target regulatory network. Based on the miRNA–target interaction network identified by the best method, we further infer miRNA–target regulatory bicliques or modules. In addition, by integrating high-confidence miRNA–target interactions and gene expression data, we identify three types of networks, including lncRNA–lncRNA, lncRNA–mRNA, and mRNA–mRNA related miRNA sponge interaction networks. To reveal the community of miRNA sponges, we further infer miRNA sponge modules from the identified miRNA sponge interaction network. Functional analysis results show that the identified hub genes, as well as miRNA-associated networks and modules, are closely linked with ASD. ASDmiR is freely available at https://github.com/chenchenxiong/ASDmiR.

## Introduction

Autism spectrum disorder (ASD) encompasses a variety of complex inheritable neurodevelopment disorders that usually occur before 3 years old and last throughout a person’s life (Fregeac et al., 2016; Quesnel-Vallieres et al., 2019). ASD patients are characterized by controlled social interactions, restricted activities, and repetitive behavior (Chen et al., 2015). The current diagnosis of ASD is mainly based on behavioral characteristics (Gillian et al., 2003), which may cause misdiagnosis or delay treatment. Previous transcriptomic studies (Voineagu et al., 2011; Gupta et al., 2014; Ansel et al., 2016; Quesnel-Vallieres et al., 2019) have reported that ASD has strong genetic complexity, and many genes are involved in the ASD-related biological processes, including neuronal activity (Voineagu et al., 2011), immune response (Gupta et al., 2014; Ansel et al., 2016), and signaling pathways (Quesnel-Vallieres et al., 2019). Although great progress has been made to study the pathogenesis of ASD, the gene regulation in ASD is largely unknown because of the heterogeneity and complexity of ASD. Therefore, it is necessary to investigate the pathogenesis and molecular mechanisms underlying ASD for improving the diagnosis and therapeutic strategies of patients.

At the genetic level, microRNAs (miRNAs) are important regulators of brain function and neuronal development (Rajman and Schratt, 2017; Shen et al., 2019). By binding with messenger RNAs (mRNAs) at the post-transcriptional level, miRNAs as tiny non-coding RNA molecules (∼22 nucleotides) can induce repression or translational inhabitation of mRNAs (Ambros, 2004). Previous studies (Ander et al., 2015; Hu et al., 2017; Shen et al., 2016) have elucidated that miRNAs participate in several biological processes that are closely associated with ASD, including synaptic plasticity and neuronal development (Hu et al., 2017), immune response (Ander et al., 2015), and signaling pathways (Shen et al., 2016). These studies have also indicated that miRNAs and their corresponding targets could help to uncover ASD pathogenesis.

Long non-coding RNAs (LncRNAs) are transcripts with a length of more than 200 nucleotides, and they play critical roles in the progression of neuropsychiatric disorders including ASD (Hosseini et al., 2019). In the developmental processes of ASD, lncRNAs take part in several important biological processes, including neuronal architecture and immune response (Kerin et al., 2012), synaptic and neuronal excitatory dysfunction (Noor et al., 2010), neurite elaboration (Wang et al., 2015), and alternative splicing (Parikshak et al., 2016). These studies have demonstrated the potential contribution of lncRNAs on revealing the molecular mechanisms of ASD.

According to competing endogenous RNA hypothesis (Salmena et al., 2011), coding and non-coding RNA transcripts compete with each other by base pairing with miRNA-recognition elements (MREs). These transcripts are also known as miRNA sponges, including mRNAs (Tay et al., 2011), lncRNAs (Cesana et al., 2011), pseudogenes (Poliseno et al., 2010), and circular RNAs (circRNAs) (Hansen et al., 2013). All types of miRNA sponges crosstalk with other through MREs and form a large-scale miRNA sponge interaction network (Salmena et al., 2011). Although accumulating miRNA sponges have been experimentally identified and are closely relevant to various cancers (Le et al., 2017), the roles of miRNA sponges in ASD are largely unknown. To uncover potential roles of miRNA sponges in ASD, we focus on investigating lncRNA and mRNA related miRNA sponge interaction networks in ASD in this work.

There have been growing computational methods to effectively explore miRNA functions based on gene expression data. However, current bioinformatics research on miRNA regulatory mechanisms related to ASD is still in its infancy. In this work, we propose a novel stepwise method, ASDmiR, to uncover miRNA regulation in ASD. ASDmiR has two main contributions as follows. First, ASDmiR can be used to study ASD-related miRNA regulation at both the network and module level. Secondly, ASDmiR can help to explore both direct and indirect miRNA regulation in ASD. At the network level, we identify two types of ASD-related networks: miRNA–target regulatory network and miRNA sponge interaction network. Meanwhile, at the module level, we infer two types of ASD-related modules: miRNA–target regulatory modules and miRNA sponge modules. Topological analysis and functional analysis have shown that the identified miRNA-associated networks and modules are highly implicated in ASD.

## Materials and Methods

### Data Acquisition and Preprocessing

#### Differential Expression Analysis

Previous studies (Mohr and Liew, 2007; Segura et al., 2015; Ansel et al., 2016) have discovered that peripheral blood samples are more accessible than brain tissue samples in the transcriptomic study of ASD. In this work, we obtained the matched miRNA, lncRNA, and mRNA expression profiles of ASD from Kong et al. (2012). The samples of gene expression profiles are from peripheral blood samples and are categorized as ASD (104 samples) and normal (82 samples). We apply the *miRBaseConverter* (Xu et al., 2018) R package to convert miRNA names into the latest version of miRBase. To discover the differentially expressed miRNAs, lncRNAs, and mRNAs between ASD samples and normal samples, we conduct differential expression analysis using the *limma* R package (Ritchie et al., 2015). In the ASD dataset, the changes in mRNA expression level between ASD samples and normal samples are large, while the changes in the case of miRNAs and lncRNAs are small. To cover important ASD-related miRNAs and lncRNAs and to have a moderate number of mRNAs for ASDmiR, we select top 100 miRNAs, 300 lncRNAs, and 4,000 mRNAs ranked by adjusted *p*-values (adjusted by the Benjamini and Hochberg method) in the differential gene expression analysis for subsequent analysis. The detailed results of differentially expressed miRNAs, lncRNAs, and mRNAs can be seen in Supplementary Table 1.

#### MiRNA-Target Interactions

For putative miRNA–mRNA interactions, we have obtained 762,540 unique interactions between 2,600 miRNAs and 21,538 mRNAs from miRTarBase v8.0 (Chou et al., 2018) and TarBase v8.0 (Karagkouni et al., 2018) databases. By combining the interactions from LncBase v2.0 (Paraskevopoulou et al., 2016) and NPInter v4.0 (Teng et al., 2020) databases, we have collected 138,951 unique miRNA–lncRNA interactions between 1,044 miRNAs and 13,243 lncRNAs. The obtained miRNA–target interactions could be seen in Supplementary Table 2.

#### ASD-Related Genes

In this work, we collect a list of miRNAs, lncRNAs, and mRNAs associated with ASD to investigate ASD-related miRNA regulation. In total, we have obtained a list of 141 ASD-related miRNAs from HMDD v3.2 (Huang et al., 2019) and MNDR v2.0 (Cui et al., 2018), a list of 117 ASD-related lncRNAs from LncRNADisease v2.0 (Bao et al., 2019) and MNDR v2.0 (Cui et al., 2018), and a list of 1,658 ASD-related mRNAs from the Simons Foundation Autism Research Initiative (SFARI)^1^ and DisGeNET v7.0 (Pinero et al., 2020). The obtained ASD-related miRNAs, lncRNAs, and mRNAs could be seen in Supplementary Table 2.

### Methods

#### Overview of ASDmiR

**FIGURE 1:**
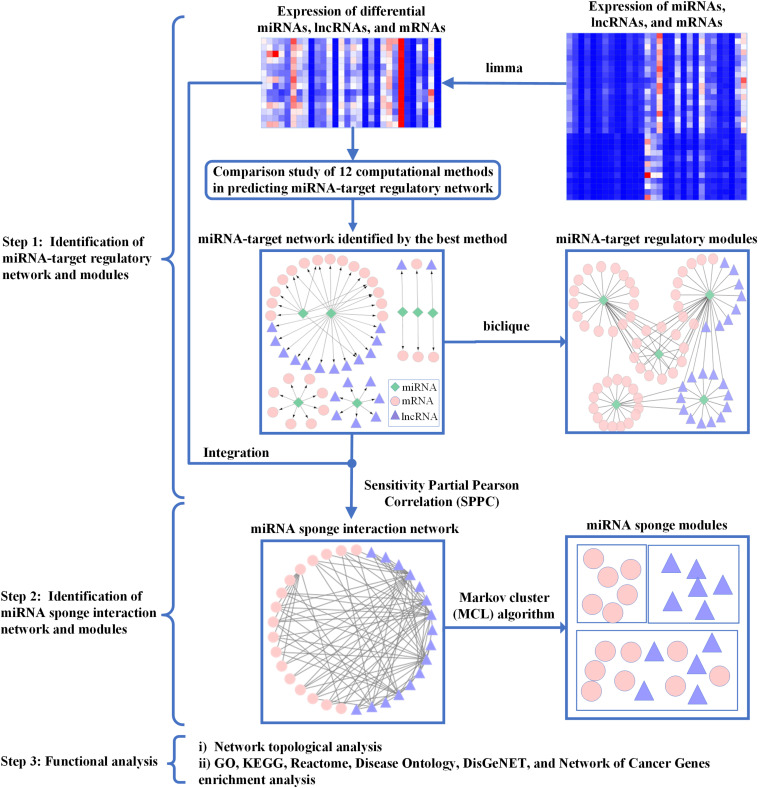
The workflow of ASDmiR. In Step 1, by integrating expression data of differential miRNAs, lncRNAs, and mRNAs and putative miRNA–target interactions, we identify miRNA–target regulatory networks using 12 existing computational methods. The miRNA–target regulatory network predicted by the best method is used for identifying miRNA–target regulatory modules. In Step 2, based on putative miRNA–mRNA interactions and gene expression data, we infer miRNA sponge interaction network using the sensitivity partial Pearson correlation method. Furthermore, we identify miRNA sponge modules from the identified miRNA sponge interaction network by using the Markov cluster algorithm. In Step 3, we conduct functional analysis of the identified miRNA-associated networks and modules. Green rhombic, pink circle, and purple triangle nodes represent miRNAs, mRNAs, and lncRNAs, respectively.

In Figure 1, the workflow of ASDmiR includes three major steps for identifying miRNA-associated networks and modules related to ASD. In the first step, by using the matched miRNA, lncRNA, and mRNA expression profiles, we conduct a comparison study of 12 commonly used miRNA target prediction methods from Le et al. (2015). In terms of the number of experimentally validated miRNA–mRNA interactions, we select the best performing method to identify miRNA–target regulatory network in ASD dataset. Furthermore, we infer miRNA–target regulatory modules based on the identified miRNA–target regulatory network. In the second step, we use the well-cited sensitivity partial Pearson correlation (SPPC) method (Paci et al., 2014) to identify miRNA sponge interaction network by integrating putative miRNA–target interactions and gene expression data. Moreover, the Markov cluster (MCL) algorithm (Enright et al., 2002) is used to discover miRNA sponge modules for investigating the community of miRNA sponges. In the final step, we conduct functional analysis of the identified miRNA-associated networks and modules. In the following, we will describe the details of these steps.

#### Identification of miRNA-Target Regulatory Network and Modules

To identify miRNA–target interactions, we use 12 existing computational methods implemented in the *miRLAB* R package (Le et al., 2015). These miRNA target prediction methods could be categorized into four types: correlation methods, regression methods, causal inference methods, and other methods. The first type of computational methods, including Pearson (Pearson, 1920), Spearman (Spearman, 1904), Kendall (Kendall, 1938), Distance correlation (Székely et al., 2007), and Hoeffding’s D measure (Hoeffding, 1948), could calculate linear correlation relationships between miRNAs and targets. To capture non-linear relationships between miRNAs and targets, the randomized dependence coefficient (Lopez-Paz et al., 2013) and Mutual Information (MI) (Moon et al., 1995) methods are utilized. For the second type of computational methods, Lasso (Tibshirani, 1996) and Elastic-net (Zou and Hastie, 2005) are used to identify the associations between miRNAs and targets. As for the third type of computational methods, the IDA (Intervention calculus when the Directed acyclic graph is Absent) method (Maathuis et al., 2009) is selected to estimate the causal effects that miRNAs have on mRNAs. For the fourth type of computational methods, *Z* score (Prill et al., 2010) and probabilistic MiRNA–mRNA Interaction Signature (ProMISe) (Li et al., 2014) are used. The *Z* score method is commonly used in gene-knockdown experiments to estimate the effect of knocking out a miRNA on mRNAs, and the ProMISe method estimates the probability of a miRNA targeting each mRNA by considering the competition among mRNAs and the competition among miRNAs. In this work, miRNAs are upstream variables, and targets (lncRNAs and mRNAs) are downstream variables. For each computational method, we use experimentally validated miRNA–target interactions as the ground truth to validate top 50, 100, 150, 200 predicted targets of each miRNA. The more the number of miRNA–target interactions validated by the ground truth is, the better the computational method performs.

It is known that genes tend to implement a specific biological process in the form of a community or module (Choobdar et al., 2019). Therefore, we further identify miRNA–target regulatory modules based on the identified miRNA–target regulatory network. Different from other biological networks (i.e., protein-protein interaction network), the miRNA–target regulatory network is a bipartite network. Consequently, the generated miRNA–target modules are actually bicliques where every miRNA of the miRNA set is connected to each target gene of the target gene set (Yoon et al., 2019). In this work, we utilize the R package *biclique* (Zhang et al., 2014) to enumerate all bicliques from the identified miRNA–target bipartite network. Here, a biclique corresponds to a miRNA–target regulatory module, and we only consider the bicliques with at least 3 miRNAs and 3 targets.

#### Identification of MiRNA Sponge Interaction Network and Modules

In this section, we apply the SPPC method (Paci et al., 2014) implemented in the *miRspongeR* R package (Zhang et al., 2019) to infer miRNA sponge interactions. The SPPC method takes miRNA, lncRNA, and mRNA expression data into account for identifying miRNA sponge interactions, and quantitatively evaluates the effect of sharing miRNAs on each miRNA sponge interaction pair at the expression level. This method uses three constraints (significant sharing of common miRNAs, significant positive correlation, and adequate sensitivity correlation) to evaluate whether a candidate RNA–RNA pair (lncRNA–lncRNA, lncRNA–mRNA, and mRNA–mRNA pair) is a miRNA sponge interaction or not. Given two competing RNAs (RNA*_*i*_* and RNA*_*j*_*), the significance *p*-value of sharing miRNAs and positive correlation is usually set to be 0.05. The Sensitive Correlation (*SC*) between the RNA*_*i*_*–RNA*_*j*_* pair is calculated as follows:

(1) $$S\,C=\rho_{i\,j}-\rho_{i\,j|n}$$

Where ρ*_*ij*_* denotes Pearson correlation (Pearson, 1920) between RNA*_*i*_* and RNA*_*j*_*, and ρ*_*ij*_*_|_*_*n*_* is partial Pearson correlation between RNA*_*i*_* and RNA*_*j*_* on the condition of *n* sharing miRNAs. In this work, the cutoff of *SC* is set to be 0.25 (see “The Identified MiRNA-Associated Modules Are Functional” for details). After assembling the identified miRNA sponge interactions, we could gain three types of networks, including lncRNA–lncRNA, lncRNA–mRNA, and mRNA–mRNA related miRNA sponge interaction networks. At the module level, we further infer miRNA sponge modules by using the Markov cluster (MCL) algorithm (Enright et al., 2002). For each module, the number of miRNA sponges (lncRNAs or mRNAs) is at least 3.

#### Functional Analysis

The hub genes may play key roles in the characteristics and development of complex diseases (Zhang et al., 2018). Consequently, at the network level, we focus on identifying hub genes from both the identified miRNA–target regulatory network and miRNA sponge interaction network. Empirically, we choose top 20% miRNAs or miRNA sponges with the largest degree as hub miRNAs or hub miRNA sponges. Furthermore, we use the miEAA (Kern et al., 2020) online tool to conduct functional enrichment analysis of hub miRNAs, and the *miRspongeR* (Zhang et al., 2019) R package for functional enrichment analysis of hub miRNA sponges.

At the module level, to know the potential diseases, biological processes, and pathways associated with the identified miRNA-associated modules, we conduct functional enrichment analysis using the well-cited *clusterProfiler* (Yu et al., 2012) R package. The third-party databases for functional enrichment analysis include Gene Ontology database (GO)^2^, Kyoto Encyclopedia of Genes and Genomes Pathway database (KEGG)^3^, Reactome Pathway database (Reactome)^4^, Disease Ontology database^5^, DisGeNET database^6^, and Network of Cancer Genes database^7^. The enriched term (GO, KEGG, Reactome, Disease Ontology, DisGeNET, or Network of Cancer Genes term) with adjusted *p* < 0.05 (adjusted by the Benjamini and Hochberg method) is regarded as a significantly enriched term.

## Results

### MiRNA-Associated Networks Are Scale-Free Networks

**FIGURE 2:**
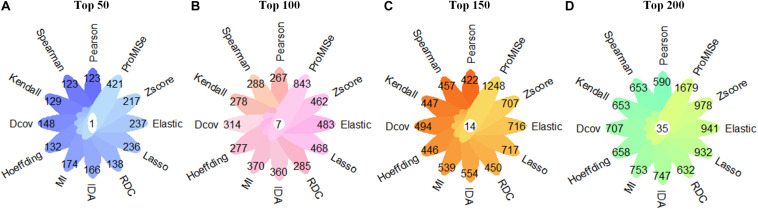
Comparison in terms of the number of confirmed miRNA–target interactions using 12 miRNA–target prediction methods. **(A)** The number of validated miRNA–target interactions in the case of top 50 miRNA–target interactions of each miRNA. **(B)** The number of validated miRNA–target interactions in the case of top 100 miRNA–target interactions of each miRNA. **(C)** The number of validated miRNA–target interactions in the case of top 150 miRNA–target interactions of each miRNA. **(D)** The number of validated miRNA–target interactions in the case of top 200 miRNA–target interactions of each miRNA. The numbers in the white circle denote the overlap of validated miRNA–target interactions by 12 computational methods.

We first follow Step 1 to obtain miRNA–target interactions predicted by each of the 12 computational methods (details in the section “Methods”). The aim of comparing the performance of these methods is to select the best prediction method to identify miRNA–target regulatory network in ASD. For each method, we select top 50, 100, 150, and 200 targets of each miRNA for the comparison. The method of predicting the largest number of experimentally validated miRNA–target interactions is used to identify miRNA–target regulatory network in ASD. As displayed in Figure 2, the ProMISe method performs the best in terms of the number of experimentally validated miRNA–target interactions. Thus, we merge top 200 targets of each miRNA identified by the ProMISe method as our final predicted miRNA–target regulatory network (consisting of 20,000 miRNA–lncRNA interactions and 20,000 miRNA–mRNA interactions). In total, we obtain a list of 1,679 validated miRNA–target interactions, consisting of 241 validated miRNA–lncRNA interactions and 1,438 validated miRNA–mRNA interactions. We further analyze the node degree distribution of the identified miRNA–target regulatory network using the Network Analyzer plugin (Assenov et al., 2008) in Cytoscape (Shannon, 2003), and discover that our identified miRNA–target regulatory network follows power law distribution well in the form of *P*(*k*) = 67.593*k*^–1.022^ with *R*^2^ = 0.783, where *P*(*k*) represents the number of nodes with the node degree *k*. A higher *R*^2^ (range from 0 to 1) indicates that the identified miRNA–target regulatory network is more likely to be a scale-free network that occurs in the real world. The detailed results of the identified miRNA–target regulatory network can be found in Supplementary Table 3.

**FIGURE 3:**
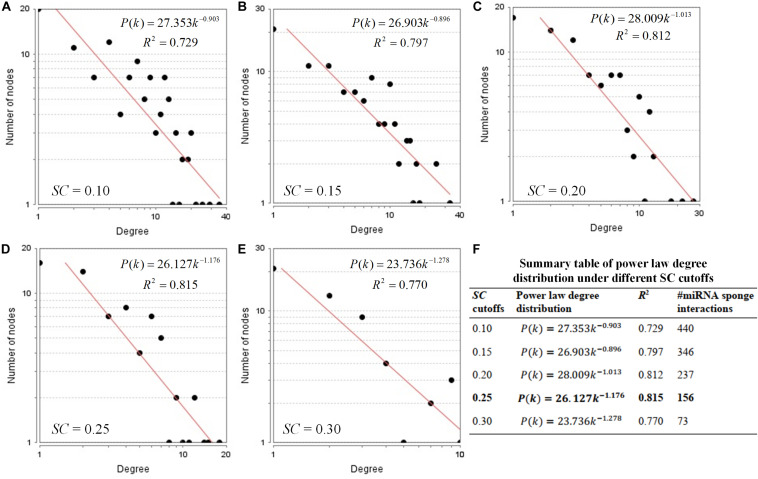
Power law degree distribution of the identified miRNA sponge interaction networks. **(A–E)** Node degree distribution of the identified miRNA sponge interaction networks using different SC cutoffs from 0.1 to 0.3 with a step of 0.05. **(F)** Summary table of power law degree distribution under different SC cutoffs.

By following Step 2, we use different *SC* cutoffs from 0.1 to 0.3 with a step of 0.05, to infer the miRNA sponge interaction network with better power law distribution. Under different *SC* cutoffs, we use *R*^2^ value to evaluate the goodness of power law degree distribution for the identified miRNA sponge interaction network. If a miRNA sponge interaction network with higher *R*^2^ value, the network is more likely to be a real biological network. As shown in Figure 3, according to the principle of the largest *R*^2^ value, we select the *SC* cutoff as 0.25 to infer miRNA sponge interaction network (containing 156 miRNA sponge interactions) that fits power law distribution well in the form of *P*(*k*) = 26.127*k*^–1.176^ with *R*^2^ = 0.815. The detailed results of the identified miRNA sponge interaction network can be found in Supplementary Table 3.

### Hub Genes Are Closely Associated With ASD

In this work, we have identified 12 hub miRNAs (*hsa-miR-195-5p*, *hsa-miR-15a-5p*, *hsa-miR-26b-5p*, *hsa-miR-23a-3p*, *hsa-miR-93-5p*, *hsa-miR-210-3p*, *hsa-miR-25-3p*, *hsa-miR-30b-5p*, *hsa-miR-148b-3p*, *hsa-miR-149-5p*, *hsa-miR-200c-3p*, and *hsa-miR-147a*) and 15 hub miRNA sponges (*SLC38A2*, *SHOC2*, *DDX6*, *WSB1*, *PURB*, *DDX5*, *DLEU2*, *USP15*, *C6orf62*, *ADAM10*, *STK4*, *LBR*, *PNISR*, *ANKRD44*, and *SERINC1*). It is noted that four hub miRNAs (*hsa-miR-148b-3p*, *hsa-miR-15a-5p*, *hsa-miR-23a-3p*, and *hsa-miR-93-5p*) and two hub miRNA sponges (*DLEU2* and *USP15*) are experimentally validated ASD-related hub genes.

**FIGURE 4:**
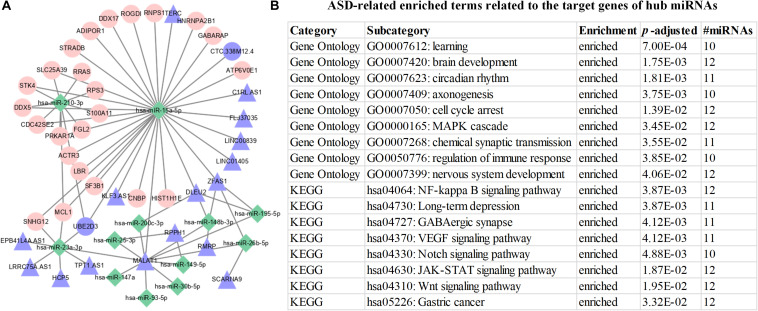
Visualization and functional enrichment analysis of hub miRNA regulatory network. **(A)** Hub miRNA regulatory network. Green rhombic, pink circle, and purple triangle nodes denote miRNAs, mRNAs, and lncRNAs, respectively. **(B)** ASD-related enriched terms related to the target genes of hub miRNAs.

In Figure 4A, we discover that 12 hub miRNAs are highly connected with their target genes, and several hub miRNAs synergistically regulate their target genes. To investigate the underlying biological implications of these hub miRNAs, we conduct functional 1enrichment analysis of the target genes of these hub miRNAs. Functional enrichment analysis results show that 439 GO terms and 128 KEGG pathways are significantly associated with the target genes of the hub miRNAs. Moreover, several significantly enriched GO biological processes and KEGG pathways, including Cell cycle arrest (GO: 0007050), Regulation of immune response (GO: 0050776) (Gupta et al., 2014) (Ander et al., 2015), Nervous system development (GO:0007399) (Hu et al., 2017), NF-kappa B signaling pathway (hsa04064) (Malik et al., 2011), Long-term depression (hsa04730) (Monday et al., 2018), Wnt signaling pathway (hsa04310) (Shen et al., 2016), and gastric cancer (hsa05226) (Wasilewska and Klukowski, 2015) are closely associated with the progression and development of ASD (Figure 4B). As for the identified hub miRNA sponges, functional enrichment analysis results indicate that they are significantly enriched in SMAD binding (GO: 0046332). A previous study (Avazzadeh et al., 2019) has demonstrated that SMAD binding is closely related to ASD. Altogether, the above functional enrichment analysis results imply that the identified hub genes are closely associated with the occurrence and development of ASD. The functional enrichment analysis results of hub genes can be found in Supplementary Table 4.

### The Identified MiRNA-Associated Modules Are Functional

Based on the identified miRNA–target network, we have identified 9,625 miRNA–target regulatory modules. In this work, we are only interested in studying the potential biological functions of top 20 largest miRNA–target regulatory modules. Moreover, we have obtained 10 miRNA sponge modules from the identified miRNA sponge interaction network. Disease and functional enrichment analysis indicate that the top 20 largest miRNA–target regulatory modules are significantly enriched in 397 GO terms, 3 KEGG pathways, 12 Reactome pathways, and 69 DisGeNET terms. Specifically, several biological processes, pathways, and diseases, including anxiety disorder (umls: C0003469) (White et al., 2009), Alternative mRNA splicing (GO: 0000380) (Quesnel-Vallieres et al., 2019), circadian rhythm (GO: 0007623) (Hu et al., 2017), and mTOR signaling pathway (R-HSA-165159) (Khlebodarova et al., 2018) are closely related to ASD (Table 1).

**TABLE 1:** Disease and functional enriched terms of top 20 largest miRNA–target regulatory modules related to ASD.

Items	Descriptions	Module ID	Evidence
umls:C1378703	Renal carcinoma	1, 8, 9, 19	Lam et al., 2018
umls:C0003469	Anxiety disorders	19	White et al., 2009
GO:0000380	Alternative mRNA splicing, via spliceosome	1, 2, 4, 6	Quesnel-Vallieres et al., 2019
GO:0007623	Circadian rhythm	1, 8	Hu et al., 2017
GO:0120111	Neuron projection cytoplasm	1, 2	Guo et al., 2019
GO:0099640	Axodendritic protein transport	1, 2	Mandal and Drerup, 2019
hsa03040	Spliceosome	5, 11, 12, 15, 16, 17, 19	Kong et al., 2013
R-HSA-210500	Glutamate neurotransmitter release cycle	5, 14	Horder et al., 2018
R-HSA-165159	mTOR signaling	5	Khlebodarova et al., 2018

Furthermore, the identified 10 miRNA sponge modules are significantly enriched in 711 GO terms, 23 KEGG pathways, 117 Reactome pathways, 22 Disease Ontology terms, and 157 DisGeNET terms. In Table 2, several GO, KEGG, Reactome, Disease Ontology, DisGeNET, and Network of Cancer Genes terms are closely associated with ASD. For instance, severe depression (umls: C0588008) (Hedley et al., 2018), Mild cognitive disorder (umls: C1270972) (Leekam, 2016), acute gastroenteritis (umls: C0267446) (Wasilewska and Klukowski, 2015), Focal adhesion (GO: 0005925) (Ander et al., 2015), long-term memory (GO: 0007616) (Toichi and Kamio, 2002), and spliceosome (hsa03040) (Kong et al., 2013) are experimentally confirmed to be ASD-related terms. Taken together, the above enrichment analysis results indicate that the identified miRNA-associated modules are functional. The disease and functional enrichment analysis results of miRNA-associated modules can be seen in Supplementary Table 5.

**TABLE 2:** Disease and functional enriched terms of miRNA sponge modules related to ASD.

Module ID	Items	Description	Adjusted *p*-value
1	GO:0061437	Renal system vasculature development	3.06E-02
	GO:0071364	Cellular response to epidermal growth factor stimulus	3.06E-02
	GO:1903844	Regulation of cellular response to transforming growth factor β stimulus	4.09E-02
	R-HSA-380972	Energy dependent regulation of mTOR by LKB1-AMPK	1.36E-02
	R-HSA-165159	mTOR signaling	1.68E-02
	R-HSA-198933	Immunoregulatory interactions between a Lymphoid and a non-lymphoid cell	4.90E-02
2	DOID:10155	Intestinal cancer	3.75E-02
	umls:C1845055	α-Thalassemia/mental retardation syndrome, non-deletion type, X-linked	2.47E-02
	GO:0000380	Alternative mRNA splicing, via spliceosome	4.23E-02
3	GO:0034134	Toll-like receptor 2 signaling pathway	3.33E-04
	GO:0002758	Innate immune response-activating signal transduction	2.82E-02
	GO:0007616	Long-term memory	2.97E-02
	GO:0000380	Alternative mRNA splicing, via spliceosome	3.71E-02
	hsa03040	Spliceosome	2.61E-02
	R-HSA-5260271	Diseases of immune system	3.03E-02
	R-HSA-1236974	ER-phagosome pathway	4.66E-02
	R-HSA-168179	Toll-like receptor TLR1:TLR2 cascade	4.66E-02
4	GO:0000784	Nuclear chromosome, telomeric region	8.97E-03
	GO:0005912	Adhere junction	1.96E-02
5	GO:0099640	Axodendritic protein transport	4.86E-02
	GO:0042754	Negative regulation of circadian rhythm	4.86E-02
	GO:1900016	Negative regulation of cytokine production involved in inflammatory response	4.86E-02
	GO:0006658	Phosphatidylserine metabolic process	4.86E-02
	GO:0002534	Cytokine production involved in inflammatory response	4.86E-02
	R-HSA-8950505	Gene and protein expression by JAK-STAT signaling after Interleukin-12 stimulation	3.64E-02
6	GO:0000783	Nuclear telomere cap complex	4.43E-02
	R-HSA-1980145	Signaling by NOTCH2	3.66E-02
	R-HSA-177929	Signaling by EGFR	3.66E-02
	R-HSA-2644603	Signaling by NOTCH1 in Cancer	3.66E-02
	umls:C0267446	Acute gastroenteritis	4.81E-02
	umls:C0588008	Severe depression	4.81E-02
7	GO:0005930	Axoneme	3.05E-02
8	umls:C0027889	Hereditary sensory and autonomic neuropathies	1.19E-02
	umls:C0235025	Peripheral motor neuropathy	1.68E-02
	umls:C0151313	Sensory neuropathy	1.93E-02
	umls:C1270972	Mild cognitive disorder	3.32E-02
	GO:0007173	Epidermal growth factor receptor signaling pathway	4.00E-02
	GO:0038127	ERBB signaling pathway	4.25E-02
	GO:0002433	Immune response-regulating cell surface receptor signaling pathway involved in phagocytosis	4.25E-02
	GO:0038094	Fc-gamma receptor signaling pathway	4.25E-02
	hsa04144	Endocytosis	8.81E-03
9	DOID:0060116	Sensory system cancer	4.63E-02
	GO:0000380	Alternative mRNA splicing, via spliceosome	8.07E-03
	GO:0007050	Cell cycle arrest	3.34E-02
	GO:0099640	Axodendritic protein transport	3.44E-02
	GO:1904357	Negative regulation of telomere maintenance via telomere lengthening	4.29E-02
	GO:0032839	Dendrite cytoplasm	4.62E-02
	GO:0005925	Focal adhesion	4.62E-02
	hsa04218	Cellular senescence	1.90E-02
	hsa03040	Spliceosome	1.37E-02
	R-HSA-9617828	FOXO-mediated transcription of cell cycle genes	3.36E-02
10	DOID:0050735	X-linked disease	1.31E-02
	GO:0005160	Transforming growth factor β receptor binding	3.18E-02
	R-HSA-2173789	TGF-β receptor signaling activates SMADs	4.82E-02
	R-HSA-2029480	Fc-gamma receptor–dependent phagocytosis	4.82E-02

## Discussion and Conclusion

Given the high prevalence rate of ASD, it becomes more and more urgent to reveal the underlying molecular mechanisms associated with ASD. Growing evidence (Fregeac et al., 2016; Shen et al., 2016; Hu et al., 2017) has revealed that miRNA dysregulation has made a great contribution to the pathology of ASD. However, there is still a lack of computational methods to uncover miRNA regulation in ASD at both the network and module level.

In this work, we propose a stepwise method, ASDmiR, to reveal miRNA regulation in ASD. The comparison study suggests that the ProMISe method is the best miRNA target prediction method for identifying miRNA–target regulatory network in ASD dataset. Network topological analysis indicates that the identified miRNA–target network and miRNA sponge interaction network are all scale-free networks. Moreover, functional enrichment analysis shows that hub miRNAs and hub miRNA sponges are closely associated with ASD. As functional units, the identified miRNA-associated modules are found to be significantly enriched in several important ASD-related terms.

ASDmiR can be improved in the following aspects. First, ASD-related samples can be obtained from peripheral blood, post-mortem brain, gastrointestinal tissue, adult olfactory stem cells, and scalp hair follicles (Ansel et al., 2016). In future, we will apply ASDmiR into other types of ASD-related datasets. Second, we will conduct a more comprehensive comparison to identify miRNA–target regulatory network by considering more miRNA target prediction methods. Third, we will cover other types of miRNA sponges (e.g., circRNAs, pseudogenes) to further uncover the potential roles of miRNA sponges in ASD. Finally, a previous study (Iakoucheva et al., 2019) has shown that *de novo* mutations (e.g., structure variants, protein-altering point mutations) and genetic variants (e.g., copy number variations, single nucleotide polymorphisms) could also contribute to the occurrence of ASD. Therefore, to further understand the molecular mechanisms of ASD, it is necessary to integrate these heterogeneous data to explore miRNA regulation.

## Data Availability Statement

The datasets presented in this study can be found in online repositories. The names of the repository/repositories and accession number(s) can be found in the article/Supplementary Material. The source codes for this study can be found at: https://github.com/chenchenxiong/ASD.

## Author Contributions

CX and JZ designed the methods. CX wrote the codes. CX, SS, and WJ analyzed the data with supervision from LM and JZ. CX, SS, and JZ wrote the manuscript. All authors read and approved the final manuscript.

## Conflict of Interest

The authors declare that the research was conducted in the absence of any commercial or financial relationships that could be construed as a potential conflict of interest.

## Footnotes

1 https://www.sfari.org/

2 http://www.geneontology.org/

3 http://www.genome.jp/kegg

4 http://reactome.org/

5 http://disease-ontology.org/

6 http://www.disgenet.org/

7 http://ncg.kcl.ac.uk/

## Supplementary Material

Supplementary Table 1 Differentially expressed miRNAs, lncRNAs, and mRNAs.

Supplementary Table 2 miRNA-target interactions and ASD-related miRNAs, lncRNAs, and mRNAs collected in relevant databases.

Supplementary Table 3 The Identified miRNA-target interactions and miRNA sponge interactions.

Supplementary Table 4 The enriched GO terms and KEGG pathways associated with hub genes.

Supplementary Table 5 The enriched disease and functional terms associated with miRNA-associated modules.
